# Association between psychological resilience and all-cause mortality in the Health and Retirement Study

**DOI:** 10.1136/bmjment-2024-301064

**Published:** 2024-08-03

**Authors:** Aijie Zhang, Liqiong Zhou, Yaxian Meng, Qianqian Ji, Meijie Ye, Qi Liu, Weiri Tan, Yeqi Zheng, Zhao Hu, Miao Liu, Xiaowei Xu, Ida K. Karlsson, Sara Hägg, Yiqiang Zhan

**Affiliations:** 1 Department of Epidemiology, School of Public Health (Shenzhen), Sun Yat-sen University, Shenzhen, Guangdong, China; 2 Department of Neurology, The Seventh Affiliated Hospital of Sun Yat-sen University, Shenzhen, Guangdong, China; 3 Department of Medical Epidemiology and Biostatistics, Karolinska Institutet, Stockholm, Sweden; 4 Institute of Environmental Medicine, Karolinska Institute, Stockholm, Sweden

**Keywords:** Cross-Sectional Studies, PSYCHIATRY, Data Interpretation, Statistical

## Abstract

**ABSTRACT:**

**Background:**

Psychological resilience refers to an individual’s ability to cope with and adapt to challenging life circumstances and events.

**Objective:**

This study aims to explore the association between psychological resilience and all-cause mortality in a national cohort of US older adults by a cross-sectional study.

**Methods:**

The Health and Retirement Study (2006–2008) included 10 569 participants aged ≥50. Mortality outcomes were determined using records up to May 2021. Multivariable Cox proportional hazards models were used to analyse the associations between psychological resilience and all-cause mortality. Restricted cubic splines were applied to examine the association between psychological resilience and mortality risk.

**Findings:**

During the follow-up period, 3489 all-cause deaths were recorded. The analysis revealed an almost linear association between psychological resilience and mortality risk. Higher levels of psychological resilience were associated with a reduced risk of all-cause mortality in models adjusting for attained age, sex, race and body mass index (HR=0.750 per 1 SD increase in psychological resilience; 95% CI 0.726, 0.775). This association remained statistically significant after further adjustment for self-reported diabetes, heart disease, stroke, cancer and hypertension (HR=0.786; 95% CI 0.760, 0.813). The relationship persisted even after accounting for smoking and other health-related behaviours (HR=0.813; 95% CI 0.802, 0.860).

**Conclusions:**

This cohort study highlights the association between psychological resilience and all-cause mortality in older adults in the USA.

**Clinical implications:**

Psychological resilience emerges as a protective factor against mortality, emphasising its importance in maintaining health and well-being.

**WHAT IS ALREADY KNOWN ON THIS TOPIC**

- Psychological resilience is the capacity for individuals to effectively cope with and adapt to challenging life circumstances and events. So far, few published studies have explored the relationship between resilience and mortality.

**WHAT THIS STUDY ADDS**

- This study is unique in establishing a statistically significant association between psychological resilience and all-cause mortality in the general population, even after accounting for confounding factors.

**HOW THIS STUDY MIGHT AFFECT RESEARCH, PRACTICE OR POLICY**

- This nationally representative study demonstrates an independent association between higher levels of psychological resilience and reduced all-cause mortality. The findings underscore the potential effectiveness of interventions aimed at promoting psychological resilience in order to mitigate mortality risks.

## Background

 Psychological resilience is the capacity for individuals to effectively cope with and adapt to challenging life circumstances and events.^1^ Exposure to adversity, such as poverty or significant life events like job loss or bereavement, often leads to disruptions in psychological functioning.^2^ However, individuals differ in their ability to overcome and adapt to adversity, with some exhibiting resilience while others struggle and experience psychological difficulties.^3^ Researchers suggest that resilience is a dynamic and active process influenced by various factors, including genetic sex, gonadal steroids and epigenetic mechanisms that regulate stress physiology. Furthermore, resilience is believed to evolve and vary across different periods.^4^

In the context of older adults, psychological resilience has shown associations with various health outcomes, including improved physical and mental well-being and successful ageing.^5^ Resilience plays a crucial role in mitigating the negative consequences of chronic illness and subsequent disability. Furthermore, high levels of resilience can protect against the detrimental effects of disability in later life.^3^ One facet of resilience, physical resilience, has been associated with the risk of mortality as individuals age. Research has delved into the connection between physical resilience and biological ageing, demonstrating that a decline in physical resilience could heighten the susceptibility to mortality, even in individuals devoid of significant morbidities.^6 7^ Building on this, there is a conjecture that psychological resilience might exhibit a comparable impact. Additionally, research has highlighted a potential association between psychological resilience and epigenetic clocks, suggesting that individuals with higher resilience may experience slower DNA methylation ageing and age acceleration.^8 9^

The relationship between psychological resilience and mortality has been examined in limited studies. Previous research has indicated the independent effects of resilience on mortality risk, even after adjusting for initial health status in a Chinese population.^10^ Additionally, improved psychological resilience has been associated with higher overall survival rates in patients with nasopharyngeal carcinoma undergoing radiotherapy.^11^ However, these findings cannot be generalised to the wider population due to their specific study samples. In a comprehensive cohort analysis of the general Italian populace, preliminary findings indicated that psychological resilience exhibited a significant relationship with all-cause mortality. However, on thorough adjustment for various covariates, the strength of this association diminished to a non-significant level.^12^ To our knowledge, few published studies have explored the relationship between resilience and mortality among the elderly in the USA.

### Objective

To address this knowledge gap, we aimed to elucidate the association between psychological resilience and mortality using the most recent data from the Health and Retirement Study (HRS) as of May 2021. We hypothesised that the higher the psychological resilience score, the lower the risk of death. Cox proportional hazards regression models were employed to estimate the magnitudes of the associations. Potential confounding factors were considered, including demographic characteristics, self-reported disease status, health-related behaviours and body mass index (BMI). Restricted cubic splines were applied to examine the association between psychological resilience and mortality risk.

## Methods

### Data source and study participants

The HRS is a nationally representative longitudinal study in the USA and includes adults over the age of 50 years together with their spouses. The study commenced in 1992 with a current sample size of almost 40 000 individuals.^13^ The study included, but was not limited to, data on the economic, health, marital and family status of older Americans. Follow-up is conducted every 2 years so that changes in the survey information of HRS participants can be tracked and recorded. For our analysis, we used two waves (2006–2008) of the HRS data. The questionnaire about psychological resilience was added for the first time in 2006. The key variables for psychological resilience were collected in the extended face-to-face interviews through the Leave-Behind Questionnaire (LBQ), which was administered in 2006 to half of the HRS participants and in 2008 to the other half. We combined both the two waves of data as the final analytical participants in the study.

The sample comprised participants who provided complete information on psychological resilience and subsequent follow-up data. A total of 19 193 participants with the LBQ were included, of which 12 527 (65.27%) provided complete psychological resilience information. Participants with missing information on mental resilience were excluded (n=6666). After excluding subjects with missing vital status (n=1948) and follow-up time data (n=10), the final sample consisted of 10 569 participants (online supplemental figure S1). All participants provided written informed consent.

### Psychological resilience

We used a simplified resilience score (SRS) that was developed based on a previous study^3^ and focused on the adjustment and management of adversity. The SRS was constructed using the 12 items of the LBQ (online supplemental table S1), guided by the five primary psychosocial domains identified in the Wagnild and Young Resilience Scale (1993).^14^ These domains encompassed qualities such as perseverance, calmness, a sense of purpose, self-reliance and the recognition that certain experiences must be faced alone. The validity of the SRS was evaluated previously,^15^ and its reliability was supported by a Cronbach’s alpha coefficient of 0.854 and substantial evidence from interitem correlation assessments.

To ensure comparability, we standardised the values of each item by dividing the respondent’s response by the maximum possible response for that item, resulting in a standardised value ranging from 0 to 1. The final psychological resilience score for each respondent was then calculated as the sum of these standardised values across all 12 items. Scores could range from 0 to 12, with higher scores indicating greater psychological resilience. To facilitate analysis, we categorised the psychological resilience scores into quartiles (Q1, Q2, Q3 and Q4), with the Q1 group serving as the reference category. Lower scores among participants indicated worse psychological resilience.

### Vital status ascertainment

The HRS cohort was followed up for mortality until May 2021. The HRS uses multiple methods to track respondents over time, including gathering information from spouses and proxy respondents to determine the vital status at each wave. It was reported that mortality ascertainment in the HRS has been effectively complete,^16^ and several studies have also applied these data to assess mortality.^17 18^ Causes of death including death due to heart, circulatory and blood conditions were also analysed.

### Covariates

It was suggested that psychological resilience shared common causes with mortality, including social environment, physical illnesses and lifestyle factors.^6^ We therefore included the following covariates in the analysis as potential confounders: demographics (sex, race and marital status), self-reported disease status (diabetes, heart disease, stroke, cancer and hypertension), health-related behaviours (smoking and physical activity) and BMI, which was calculated in kilogram per square metre. Race was categorised as White/Caucasian, Black/African, or Others, while marital status was classified as married, single, separated/divorced and widower. Smoking was divided into current smokers and non-smokers. Physical activity was classified as more than once a week, less than once a week and no exercise. The proportions of missing values for covariates including BMI, diabetes, heart disease, stroke, cancer and hypertension were only minor (0.08–1.5%). We deleted those with missing values in the models for these covariates.

### Statistical analysis

Analyses were performed on 10 569 individuals that were grouped in quartiles of psychological resilience with about 2640 participants in each quartile. The incidence rates of all-cause mortality for each psychological resilience quartile group were calculated. Cox proportional hazards regression models were employed to estimate the magnitudes of the associations. Attained age was used as the underlying timescale. Due to the significant sex differences in psychological resilience, we stratified analysis by sex. A proportional hazards assumption based on Schoenfeld residuals was tested. If the proportional hazards hypothesis was violated, stratified Cox regression analysis by follow-up time was performed. In this study, we constructed multiple successive models based on sociodemographics, disease status and individual behavioural factors to minimise confounding bias and investigate if a single confounder could affect the results significantly. To investigate the potential non-linear relationship between psychological resilience and all-cause mortality, Cox proportional hazards regression models with restricted cubic splines were conducted. We used *survival*, *survminer*, *ggplot2* and *rms* packages. A statistically significant level is set at 0.05 and two-tailed tests were used. Statistical analyses were performed with R V.4.2.1 (R Core Team, Vienna, Austria).

## Findings

The characteristics of the study participants are shown in table 1. Of the 10 569 participants, the mean chronological age was 66.95 years and 58.84% were female. The average psychological resilience score of the study sample was 9.18, in which the psychological resilience score of men was 9.19±1.69, and that of women was 9.17±1.80 (p=0.50).

**Table 1:** Descriptive measures of participant characteristics, HRS 2006–2008, n=10 569

Characteristics	Total (n=10 569)		Men (n=4350)		Women (n=6219)	
	Mean	SD	Mean	SD	Mean	SD
Age (years)	66.95	10.22	67.50	9.54	66.57	10.66
BMI (kg/m^2^)	33.44	7.73	7.31	7.31	7.90	7.90
Resilience score	9.18	1.75	9.19	1.69	9.17	1.80
	n	%	n	%	n	%
Race						
White/Caucasian	8871	84.20	3746	86.41	5125	82.65
Black/African American	1239	11.76	438	10.10	801	12.92
Others	426	4.04	151	3.48	275	4.43
Marital status						
Married	6986	66.10	3423	78.69	3563	57.29
Single	342	3.24	143	3.29	199	3.20
Separated/divorced	1309	12.39	437	10.05	872	14.02
Widower	1932	18.28	347	7.98	1585	25.49
Diabetes						
Yes	1725	16.50	775	18.01	950 (15.44)	950 (15.44)
No	8731	83.50	3528 (81.99)	3528 (81.99)	5203	84.56
Heart problems						
Yes	2236	21.39	1111	25.81	1125	18.29
No	8219	78.61	3193	74.19	5026	81.71
Stroke						
Yes	501	4.79	245	5.69	256	4.16
No	9959	95.21	4061	94.31	5898	95.84
Cancer						
Yes	1315	12.58	560	13.01	755	12.27
No	9139	87.42	3743	86.99	5396	87.73
Hypertension						
Yes	6074	57.52	2480	57.04	3594	57.86
No	4486	42.48	1868	42.96	2618	42.14
Current smoker						
Non-smoker	1376	13.09	592	13.70	784	12.67
Smoker	9134	86.91	3728	86.30	5406	87.33
Physical activity						
>Once a week	6048	57.26	2665	61.29	3383	54.43
≤Once a week	2497	23.64	1055	24.26	1442	23.20
Hardly ever	2018	19.10	628	14.44	1390	22.37

BMI, body mass index; HRS, Health and Retirement Study.

**Figure 1:**
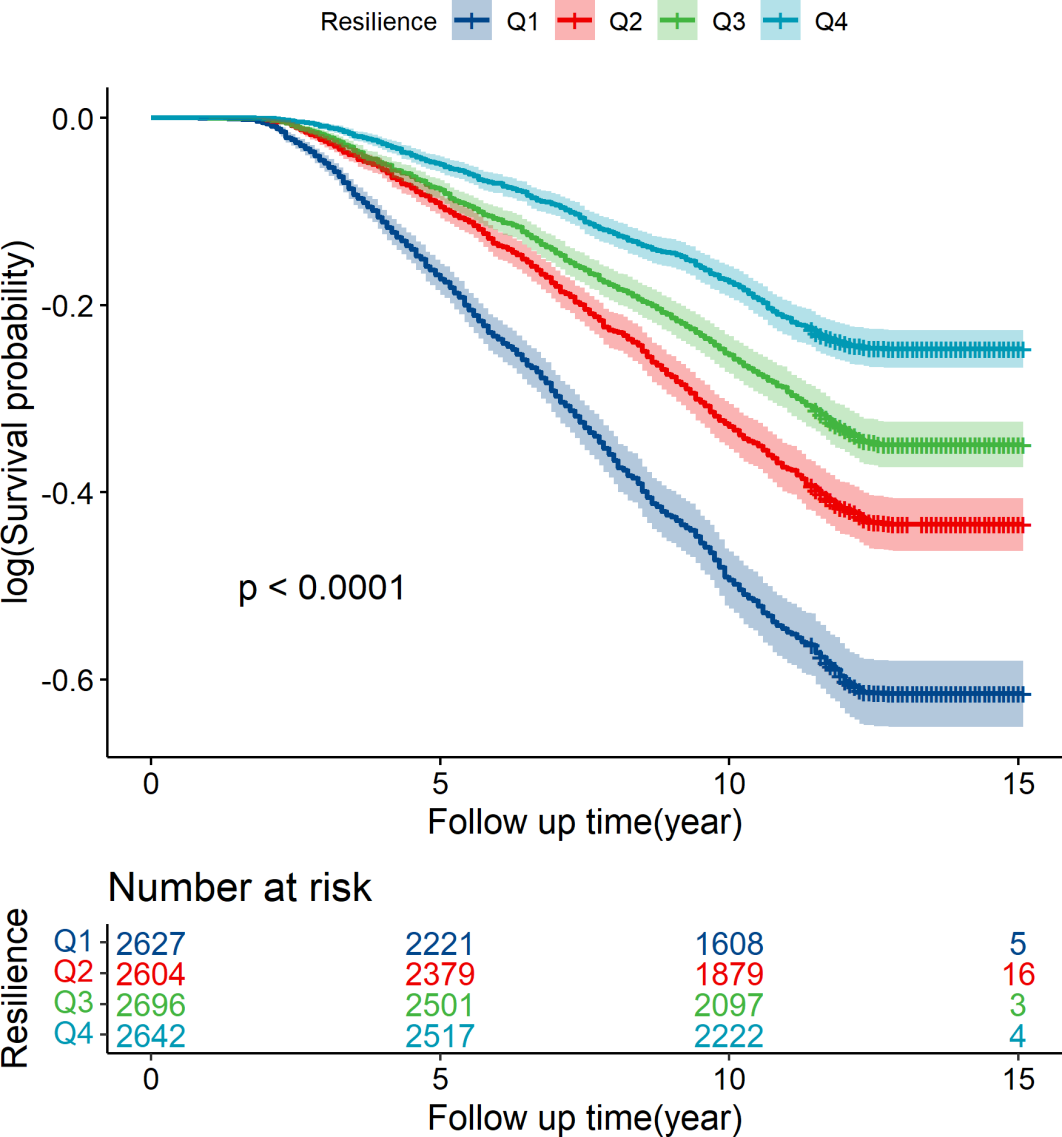
Kaplan-Meier survival curves for all-cause death during follow-up, Health and Retirement Study (HRS) 2006–2021, n=10 569.

Over a mean follow-up time of 11.6 years and a median follow-up time of 12.3 years, 3489 all-cause deaths were recorded. We ranked psychological resilience scores into quartiles, and their 10-year survival probabilities were 61.0% (Q1), 71.9% (Q2), 77.7% (Q3) and 83.9% (Q4). The survival curve and cumulative risk table were made according to the psychological resilience quartile groups (figure 1), and the difference between groups was statistically significant (p=4.15e-89).

The survival analysis (table 2) showed that in model 1, participants with the highest quartile of psychological resilience had a 53% lower risk of all-cause mortality compared with the first quartile (HR=0.470; 95% CI 0.426, 0.520; p<0.0001). This association remained statistically significant after adjusting for demographic status, including sex, race and BMI in model 2 (HR=0.473; 95% CI 0.428, 0.524; p<0.0001).

**Table 2:** HRs for all-cause mortality and psychological resilience (quartiles), HRS 2006–2008, n=10 569; HR (95% CI)

Psychological resilience quartiles	n	Deaths (n)	Person-years	Model 1	Model 2	Model 3	Model 4
Q1 (1.93, 8.05)	2627	1205	27 510.27	1	1	1	1
Q2 (8.07, 9.38)	2604	916	30 003.10	0.687 (0.630, 0.748)	0.685 (0.628, 0.747)	0.742 (0.679, 0.810)	0.798 (0.731, 0.873)
Q3 (9.40, 10.55)	2696	791	32 121.53	0.593 (0.541, 0.648)	0.588 (0.537, 0.644)	0.655 (0.597, 0.718)	0.732 (0.667, 0.804)
Q4 (10.57, 12.00)	2642	577	33 070.43	0.470 (0.426, 0.520)	0.473 (0.428, 0.524)	0.538 (0.485, 0.596)	0.619 (0.557, 0.688)
Continuous (per 1 SD)	10 569	3489	122 705	0.754 (0.730, 0.779)	0.750 (0.726, 0.775)	0.786 (0.760, 0.813)	0.831 (0.802, 0.860)

Model 1: attained age.

Model 2: additionally adjusting for sex, race and body mass index (BMI).

Model 3: additionally adjusting for diabetes, heart disease, stroke, cancer and hypertension.

Model 4: additionally adjusting for smoking, physical activity and marital status.

HRS, Health and Retirement Study.

The inclusion of baseline disease status in model 2 attenuated the association between psychological resilience and the risk of all-cause mortality (HR=0.538; 95% CI 0.485, 0.596; p<0.0001). When smoking, exercise and marital status were additionally added to model 3, the magnitudes of the associations in model 4 were further decreased compared with model 3 and showed a downward trend with the psychological resilience score (HR=0.619; 95% CI 0.557, 0.688; p<0.0001). A statistically significant association between psychological resilience and all-cause mortality was observed in all models.

We constructed stratified Cox regression models by stratifying follow-up time because the proportional hazards assumptions were not satisfied for psychological resilience. The HR decreased compared with the overall sample at a follow-up time of less than 9 years (online supplemental table S2), but increased at a follow-up time of more than 9 years (online supplemental table S3). In the above-stratified analysis, the association between psychological resilience and the risk of death remained statistically significant. The association of psychological resilience with cardiovascular mortality was also examined and the results showed a general agreement with all-cause mortality (online supplemental table S4, HR=0.852; 95% CI 0.805, 0.901; p=2.54e-08).

**Figure 2:**
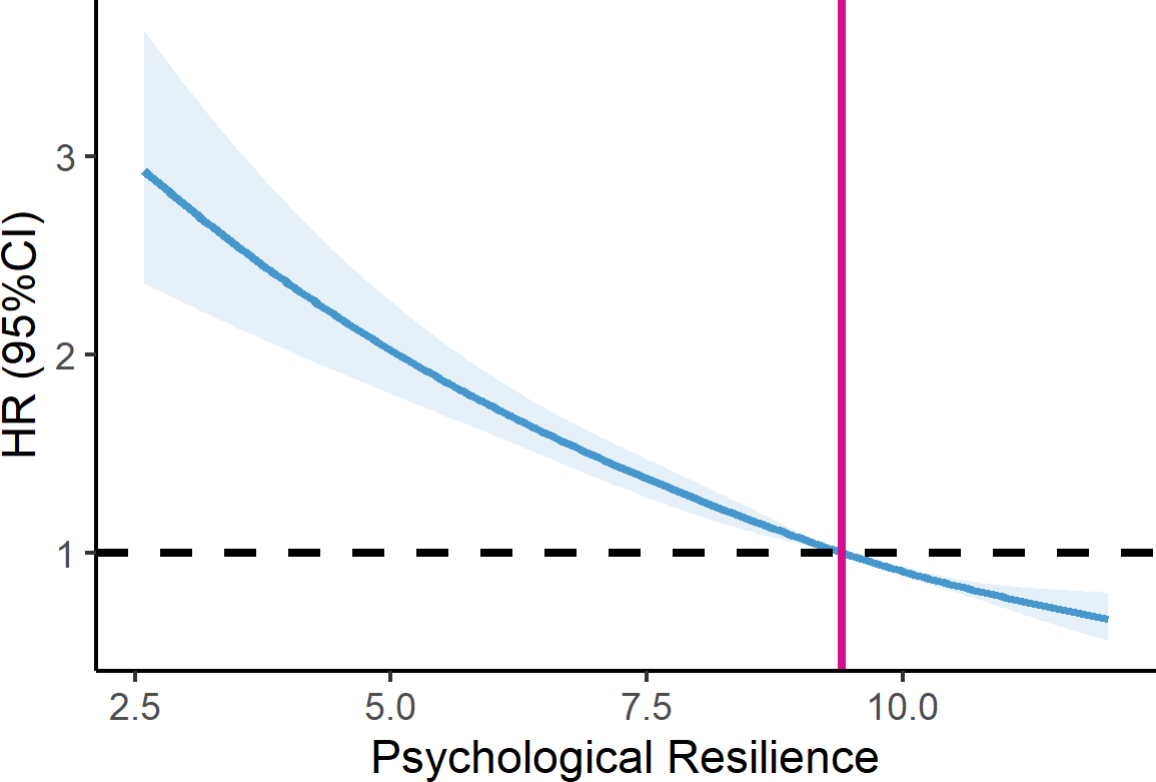
Association between psychological resilience and mortality risk in Health and Retirement Study (HRS), HRS 2006–2008, n=10 569. (Each HR was computed with a psychological resilience score level of 9.4 as the reference. Adjusted for age, sex, race, diabetes, heart disease, stroke, cancer, hypertension, smoking, physical activity and body mass index. The solid line and blue area represent the estimated values and their corresponding 95% CI.)

**Figure 3:**
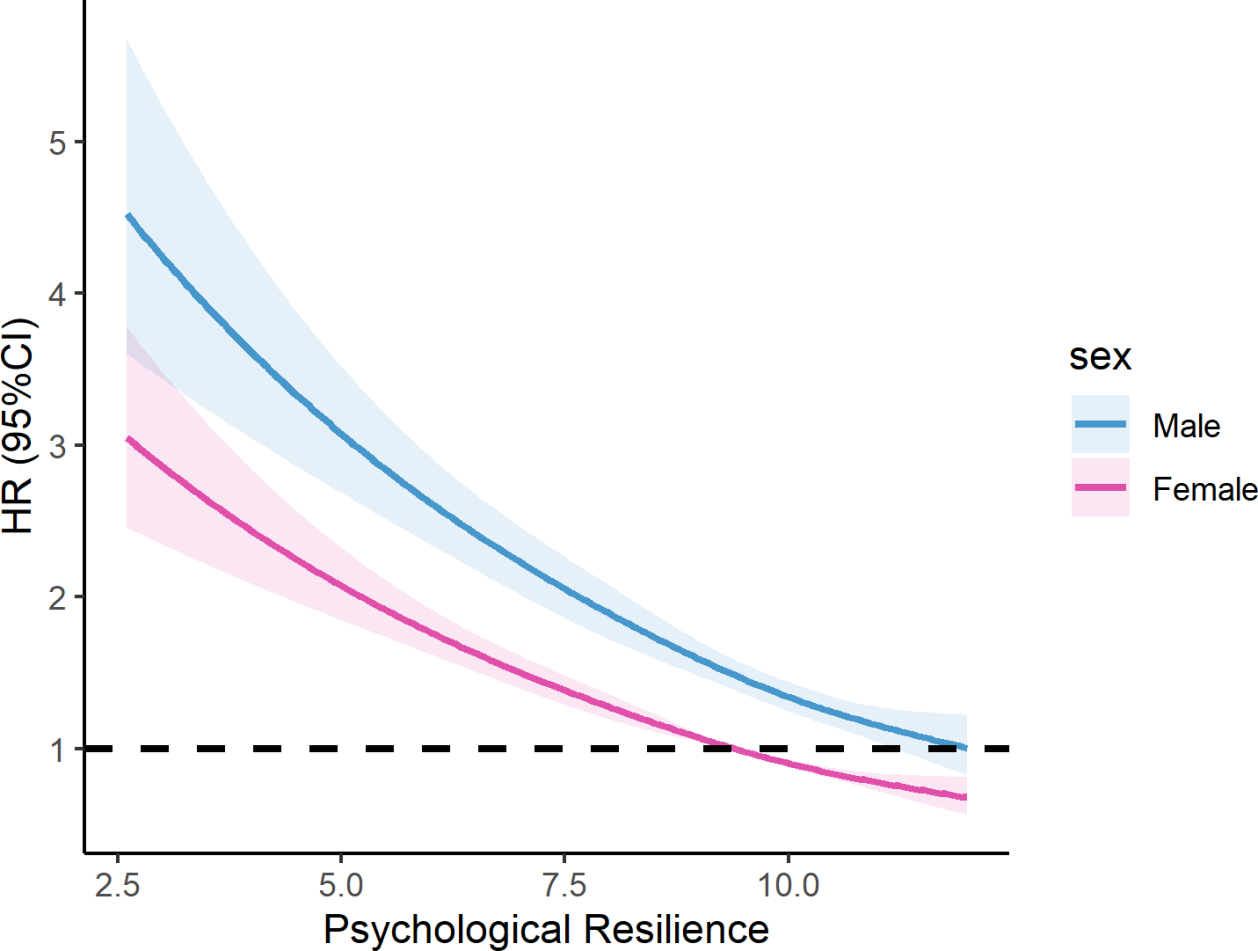
Association between psychological resilience and mortality risk by sex in Health and Retirement Study (HRS), HRS 2006–2008, n=10 569.

The psychological resilience score and the all-cause mortality exhibited an almost linear relationship (p for non-linear=0.94). In the overall population, the higher the psychological resilience score, the lower the observed mortality risk ratio (figure 2). Stratifying the fitted curve by sex, figure 3 shows that women have a lower HR than men.

## Discussion

In this study, we examined the impact of psychological resilience on all-cause mortality in a sample of 10 569 participants from the HRS. Our findings revealed a protective effect of psychological resilience against mortality. After adjusting for potential confounding factors, we observed a decrease in the risk of death by 20.2% (Q2), 26.8% (Q3) and 38.1% (Q4) in the three groups with higher psychological resilience scores, compared with the group with the lowest scores (Q1) with a median follow-up time of 12.3 years.

Our study contributes to the existing literature on the relationship between psychological resilience and all-cause mortality, consistently supporting previous findings.^10^,^12^ However, it is worth noting that research in this area remains scarce, particularly in general populations. Previous studies have mostly focused on specific populations, such as older adults or patients with specific medical conditions. For instance, a cohort study involving older adults found that better resilience was associated with a decreased risk of mortality, with a larger effect observed among the youngest old.^10^ However, the study only used demographic data and could be biased by residual confounding. The HRS database used in our study contains a large amount of mental health-related information, rending us to perform a more detailed analysis. Another study on patients with nasopharyngeal carcinoma demonstrated an association between improved psychological resilience and higher overall and progression-free survival rates.^11^ This study, however, was limited to a specific group of people with cancer. Our study population was not limited to participants with specific diseases and the results could be generalised. To our knowledge, the only study investigating the link between psychological resilience and all-cause mortality in the general population was conducted in the Moli-Sani Study, which included 10 406 participants.^12^ Similar to our findings, this study reported a 20% reduction in all-cause mortality associated with higher psychological resilience (follow-up duration: 11.2 years). However, compared with our study, which maintained significance after adjusting for numerous covariates, the statistical significance of this study disappeared after variable adjustment. Additionally, factor analysis and subsequent Cox regression in that study revealed that *positive acceptance of change*, a core aspect of psychological resilience, was associated with lower overall mortality and reduced risk of all-cause and cardiovascular death among older participants. Our study also found that higher resilience scores were associated with lower cardiovascular mortality. This suggests that individuals with a more positive self-perception and greater acceptance of change tend to have better functional health and promote longevity.^19^

Resilience is often discussed in terms of protective factors, allowing adults in normal environments to maintain relative stability even in the face of highly disruptive events. While they may experience changes such as insomnia and inattention, their overall physiological and psychological functions remain normal or close to normal. Additionally, they can even experience positive emotions following trauma.^20^ Various factors, including but not limited to meaning in life, positive emotions, self-rated health and satisfaction with social support, have been identified as potential influences on psychological resilience. Triggering these positive emotions may enhance the protective effects of psychological resilience and mitigate the negative impact of accumulated adversity on mental health in adults.^21^ Specifically, there are data that demonstrated older adults with a strong sense of meaning in life were less likely to die compared with those without such a sense, with a stronger association observed for the dimension of having a strong sense of purpose in life.^22^ Many older adults face multiple chronic conditions, which often exacerbate the inflammatory process and lead to functional decline. However, this effect was alleviated among older adults who had higher levels of life purpose (as a component of psychological resilience) and positive relationships with others.^23^ Furthermore, recent studies have shown a significant positive correlation between life goals and self-rated health, with life goals moderating the relationship between self-rated health and mortality. Poor self-rated health consistently predicts shorter life expectancy, even when objective disease conditions and risk factors are considered. On the other hand, maintaining a positive self-perception of ageing has a considerable effect on functional health, and optimism independently protects against all-cause mortality.^19 24 25^ Prospective studies and reviews consistently demonstrate that individuals with a low number or poor quality of social relationships have an increased risk of death, while positive social support contributes to successful ageing.^26 27^ These essential factors collectively contribute to psychological resilience and may explain its association with longevity. Furthermore, the ability to overcome negative emotional disturbances is also a crucial aspect of resilience and may contribute to the reduction in mortality from multiple dimensions.

This study is unique in establishing a statistically significant association between psychological resilience and all-cause mortality in the older and retired population, even after accounting for confounding factors. Key strengths of the study include its prospective design, large sample in a general setting, extensive follow-up period of over 10 years and adjustment for various lifestyle and baseline health conditions, thereby enhancing the credibility of the findings. However, certain limitations should be acknowledged. First, while the items in our SRS closely align with the Wagnild and Young scales, some items may not fully correspond. Because SRS is a questionnaire, it does not capture the dynamic nature of psychological resilience as an assessment tool. By missing the dynamic aspect, our results may only show part of the possible impact of psychological resilience on health and disease. Future research could explore the broader impacts of psychological resilience on health using alternative scales and analyse potential moderating effects through individual resilience items. Second, as an observational study, it is important to note that causality cannot be inferred. Despite controlling for numerous covariates, the influence of unmeasured variables on the results cannot be excluded. For example, genetic factors, epigenetic regulation, organisational effects of hormones and childhood adversity were not considered in our analysis.^4^ Additionally, the analysis solely relies on baseline data, potentially overlooking changes during follow-up that could affect outcomes.

### Clinical implications

In conclusion, this nationally representative study demonstrates an independent association between higher levels of psychological resilience and reduced all-cause mortality. The findings underscore the potential effectiveness of interventions aimed at promoting psychological resilience in order to mitigate mortality risks.

## Supplementary material

10.1136/bmjment-2024-301064 online supplemental file 1

## Data Availability

Data are available in a public, open access repository.
