# Supplementary material for: Association between psychological resilience and all-cause mortality in the Health and Retirement Study
Source: BMJ Ment Health. 2024 Aug 3;27(1):e301064. doi: 10.1136/bmjment-2024-301064 (PMC11409260; doi:10.1136/bmjment-2024-301064)

Supplementary Tables and Figures

Table S1 Twelve-Item Simplified Psychological Resilience Score.

Table S2 Hazard Ratios for All-cause Mortality and Psychological Resilience (quartiles), HRS 2006-2008 (follow-up time< 9 years), N=10,584.

Table S3 Hazard Ratios for All-cause Mortality and Psychological Resilience (quartiles), HRS 2006-2008 (follow-up time≥ 9 years), N=8,272.

Table S4 Hazard Ratios for Cardiovascular Mortality and Psychological Resilience (quartiles), HRS 2006-2008, N=10,569; Hazard Ratio (95% Confidence interval).

Fig. S1 Flow Chart of Participants Selection.

Table S1 Twelve-Item Simplified Psychological Resilience Score

Resilience items
1. I feel it is impossible for me to reach the goals that I would like to strive for*
2. So far, I have gotten the important things I want in life
3. If something can go wrong for me, it will*
4. I am satisfied with my life
5. I feel that what happens in life is often determined by factors beyond my control*
6. I can do the things that I want to do
7. The future seems hopeless to me and I can't believe that things are changing for the better*
8. When I really want to do something, I usually find a way to succeed at it
9. In most ways, my life is close to ideal
10. I can do just about anything I set my mind to
11. There is really no way I can solve the problems I have*
12. I have a sense of direction and purpose in life

* Health and Retirement Study items were reverse coded to make them comparable to the Wagnild and Young items.

Table S2 Hazard Ratios for All-cause Mortality and Psychological Resilience (quartiles), HRS 2006-2008 (follow-up time< 9 years), N=10,569.

	**Q1** HR (95% Cl)	**Q2** HR (95% Cl)	**Q3** HR (95% Cl)	**Q4** HR (95% Cl)	**P value for trend**
N	2,627	2,604	2,696	2,642	
N of deaths	909	625	510	354	
Person-years	20398.01	21455.22	22582.43	22684.59	
Model 1	1	0.668 (0.603, 0.740)	0.571 (0.512, 0.637)	0.450 (0.398,0.510)	**<2e-16***
Model 2	1	0.672 (0.606, 0.745)	0.573 (0.514, 0.640)	0.461 (0.406, 0.523)	**<2e-16***
Model 3	1	0.720 (0.649, 0.800)	0.632 (0.565, 0.706)	0.516 (0.454, 0.587)	**<2e-16***
Model 4	1	0.772 (0.694, 0.858)	0.701 (0.626, 0.785)	0.590 (0.518, 0.673)	**<2e-16***

Follow-up time = last age – entry age; HR: Hazard Ratio

Model 1: no covariates;

Model 2: Model 1 + sex, race, BMI;

Model 3: Model 2 + diabetes, heart disease, stroke, cancer, hypertension;

Model 4: Model 3 + smoking, physical activity, marital status.

**P* Value < 0.05 (two-sided)

Table S3 Hazard Ratios for All-cause Mortality and Psychological Resilience (quartiles), HRS 2006-2008 (follow-up time≥ 9 years), N=8,272.

	Q1	Q2	Q3	Q4	P value for trend
	**HR (95% Cl)**	**HR (95% Cl)**	**HR (95% Cl)**	**HR (95% Cl)**	
N	1,721	1,983	2,189	2,291	
N of deaths	296	291	281	223	
Person-years	22601.26	26394.88	29240.1	31004.84	
Model 1	1	0.785<br>(0.667 0.923)	0.714<br>(0.606, 0.841)	0.630<br>(0.529,0.751)	**1.20e-07***
Model 2	1	0.778<br>(0.660, 0.917)	0.702<br>(0.595, 0.829)	0.631<br>(0.528, 0.754)	**1.82e-07***
Model 3	1	0.831<br>(0.704, 0.980)	0.762<br>(0.644, 0.902)	0.690<br>(0.576, 0.827)	**3.36e-05***
Model 4	1	0.885<br>(0.749, 1.045)	0.832<br>(0.702, 0.987)	0.771<br>(0.642, 0.927)	**4.27e-03***

Follow-up time = last age – entry age; HR: Hazard Ratio

Model 1: no covariates;

Model 2: Model 1 + sex, race, BMI;

Model 3: Model 2 + diabetes, heart disease, stroke, cancer, hypertension;

Model 4: Model 3 + smoking, physical activity, marital status.

**P* Value < 0.05 (two-sided)

Table S4 Hazard Ratios for Cardiovascular Mortality and Psychological Resilience (quartiles), HRS 2006-2008, N=10,569; Hazard Ratio (95% Confidence interval).

Psychological Resilience Quartiles	N	N of deaths	Person-years	Model 1	Model 2	Model 3	Model 4
**Q1(1.93,8.05)**	2,627	453	27,510.27	1	1	1	1
**Q2(8.07,9.38)**	2,604	319	30,003.10	0.665(0.575, 0.768)	0.680(0.587, 0.787)	0.767(0.661, 0.890)	0.823(0.708, 0.955)
**Q3(9.40,10.55)**	2,696	272	32,121.53	0.572(0.492, 0.666)	0.585(0.501, 0.682)	0.681(0.582, 0.796)	0.758(0.647, 0.888)
**Q4(10.57,12.00)**	2,642	182	33,070.43	0.421(0.354, 0.502)	0.434(0.363, 0.518)	0.528(0.440, 0.633)	0.597(0.496, 0.718)
**Continuous (per 1 SD)**	10,569	1,226	122,705	0.755(0.716, 0.797)	0.763(0.722, 0.806)	0.816(0.772, 0.862)	0.852(0.805, 0.901)

Model 1: attained age;

Model 2: additionally adjusting for sex, race, and BMI;

Model 3: additionally adjusting for diabetes, heart disease, stroke, cancer, and hypertension;

Model 4: additionally adjusting for smoking, physical activity, and marital status.

Fig. S1 Sample screening flow chart

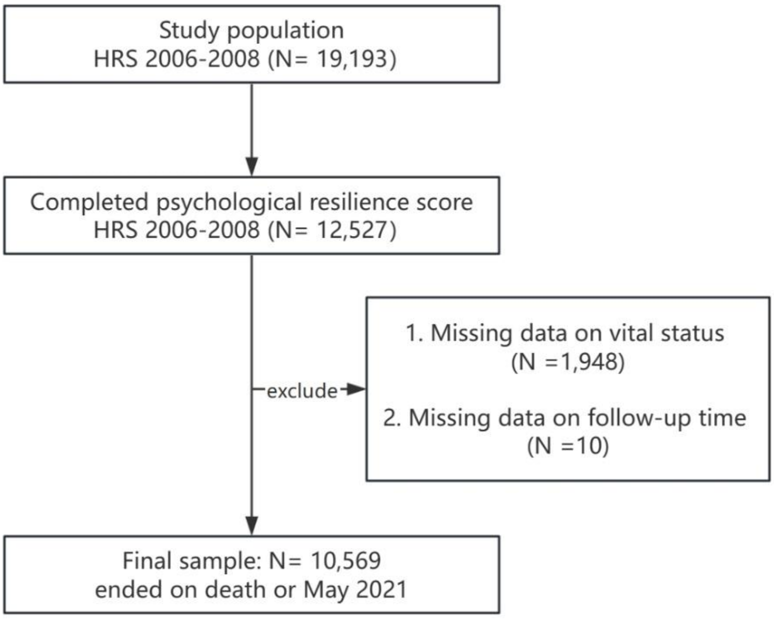

Supplement: online supplemental file 1 [file bmjment-27-1-s001.pdf]
